# Differentiated Papillary NUT Carcinoma: An Unexpected, Deceptively Bland Presentation of a Sinonasal Carcinoma

**DOI:** 10.1007/s12105-023-01554-w

**Published:** 2023-04-28

**Authors:** Martin Wartenberg, Sara-Lynn Hool, Andrea Marrazzini, Roland Giger, Niels J. Rupp

**Affiliations:** 1 Institute of Tissue Medicine and Pathology, University of Bern, Murtenstrasse 31, CH-3008 Bern, Switzerland; 2 Department of Otorhinolaryngology, Head and Neck Surgery, Inselspital, Bern University Hospital, and University of Bern, Bern, Switzerland; 3 Department of Pathology and Molecular Pathology, University Hospital Zurich, Zurich, Switzerland; 4 Faculty of Medicine, University of Zurich, Zurich, Switzerland

**Keywords:** NUT carcinoma, *DEK::AFF2* carcinoma, EBV- and HPV-associated carcinomas, Undifferentiated as well as SWI/SNF complex deficient sinonasal carcinomas

## Abstract

**Background:**

In recent years, the list of tumor entities in the sinonasal tract has significantly expanded, requiring advanced diagnostic testing. We report the case of a 32-year-old patient with an unusual NUT carcinoma originating in the maxillary sinus, which showed extensive well-differentiated, papillary squamous morphology, similar to the spectrum of the recently described *DEK::AFF2* fusion-associated carcinoma.

**Methods:**

We performed immunohistochemical and molecular studies including EBV- and HPV-testing, as well as DNA/RNA next generation sequencing.

**Results:**

The tumor showed predominantly exophytic papillary growth with mature squamous differentiation. An additional component harbored atypical, less differentiated basaloid tumor cells with infiltration of the adjacent stroma. Conspicuous inflammation was evident. There was no evidence of HPV DNA or EBV RNA. Next-generation sequencing revealed a *NUT::NSD3* gene fusion corresponding to (“speckled-type”) immunopositivity of NUT in the tumor cells.

**Conclusions:**

We describe a *NUT::NSD3* gene fusion-associated NUT carcinoma of the sinonasal tract with a deceptively well-differentiated papillary growth pattern, thus expanding the morphological spectrum of this typically poorly differentiated neoplasm.

## Introduction

Over the past decade, the spectrum of malignant tumors of the head and neck has expanded, with many entities characterized by distinct molecular alterations. For example, carcinomas comprise conventional squamous cell carcinomas, NUT carcinomas, *DEK::AFF2* carcinomas, EBV- and HPV-associated carcinomas, undifferentiated as well as SWI/SNF complex deficient sinonasal carcinomas, highlighting the variety of different morphologies and molecular pathogeneses [1]. However, morphological overlap between different entities should be considered during the process of histopathological diagnosis. Here we report the case of a 32-year-old male patient, who presented with a non-healing lesion of the upper alveolar ridge after tooth extraction, leading to an oro-antral fistula. The histological features of the initial biopsy appeared deceptively bland, prompting the differential diagnosis of reactive inflammatory changes. However, an external histopathological consultation accompanied by molecular work-up with the detection of a *NSD3::NUTM1* fusion, yielded the unexpected diagnosis of a sinonasal NUT carcinoma originating from the maxillary sinus.

### Clinical Presentation

**Fig. 1:**
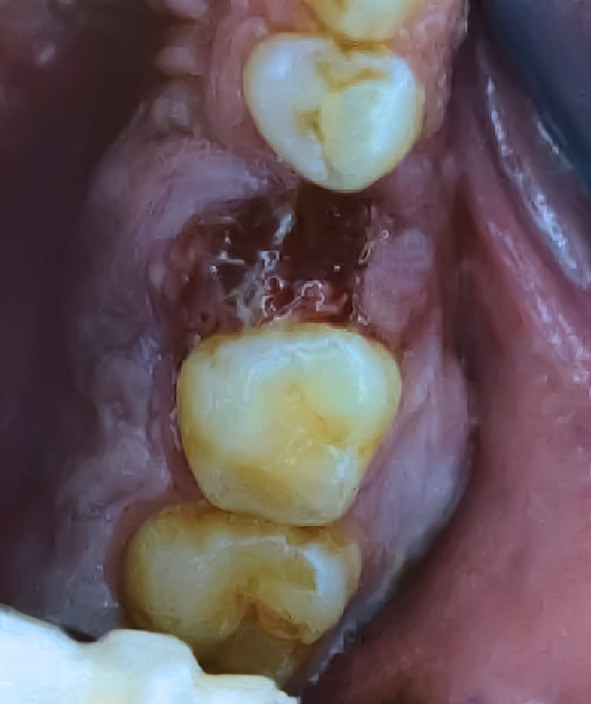
Oro-antral fistula on the alveolar ridge of the left upper jaw after tooth extraction 25

**Fig. 2:**
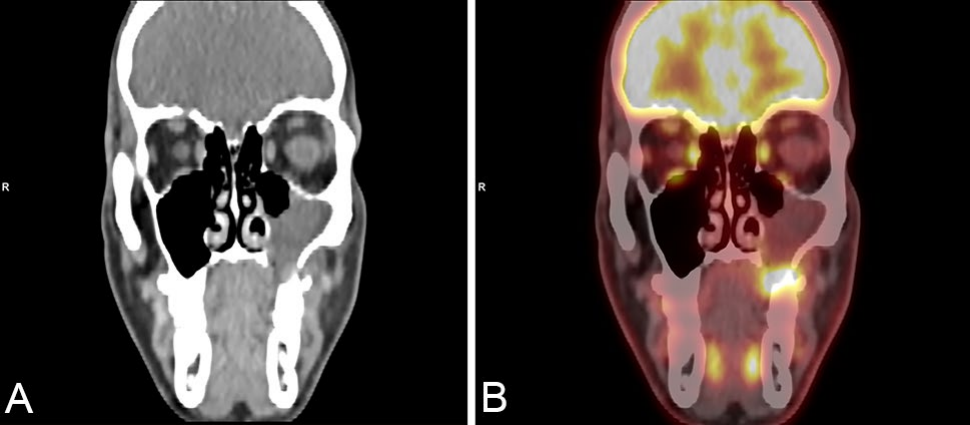
**A** Coronal CT scan of the paranasal sinuses showing a large bone defect and opacification of the left maxillary sinus. **B** FDG-PET/CT showing an intensely metabolically active tumor with osseous destruction of the left maxillary sinus

A 32-year-old, otherwise healthy, actively smoking male patient, presented with a 5-month history of pain in the left upper jaw. He was referred to a dentist, and after treatment, including extraction of tooth 25, wound healing delay was accompanied by a persistent oro-antral fistula (Fig. 1). On examination, the fistula was localized at the site of the extracted tooth and the alveolar ridge of the posterior part appeared enlarged. The first biopsy showed chronic-active inflammatory changes. A post-biopsy CT scan of the paranasal sinuses demonstrated a large osseous defect and bone erosion at the site of the extracted tooth with complete opacification of the left maxillary sinus (Fig. 2A). Despite the inflammatory changes noted in the first biopsy, the CT scan was interpreted as suspicious for malignancy. A second, larger biopsy was performed one month later, revealing a non-keratinizing squamous cell carcinoma. For staging purposes and resection planning, a whole body FDG-PET/CT was performed, showing metabolically enhanced osseous destruction in the left maxillary sinus (Fig. 2B). The patient was discussed at our multidisciplinary tumor board with the consensus that disease was staged as cT2 cN0 cM0 (UICC/TNM 8^th^ edition), requiring primary resection. A hemimaxillectomy with wide margins was performed. In addition, the patient underwent a selective neck dissection level I-III on the left side followed by reconstruction of the defect with a superficial circumflex iliac artery-based iliac bone-free flap.

### Pathology

**Fig. 3:**
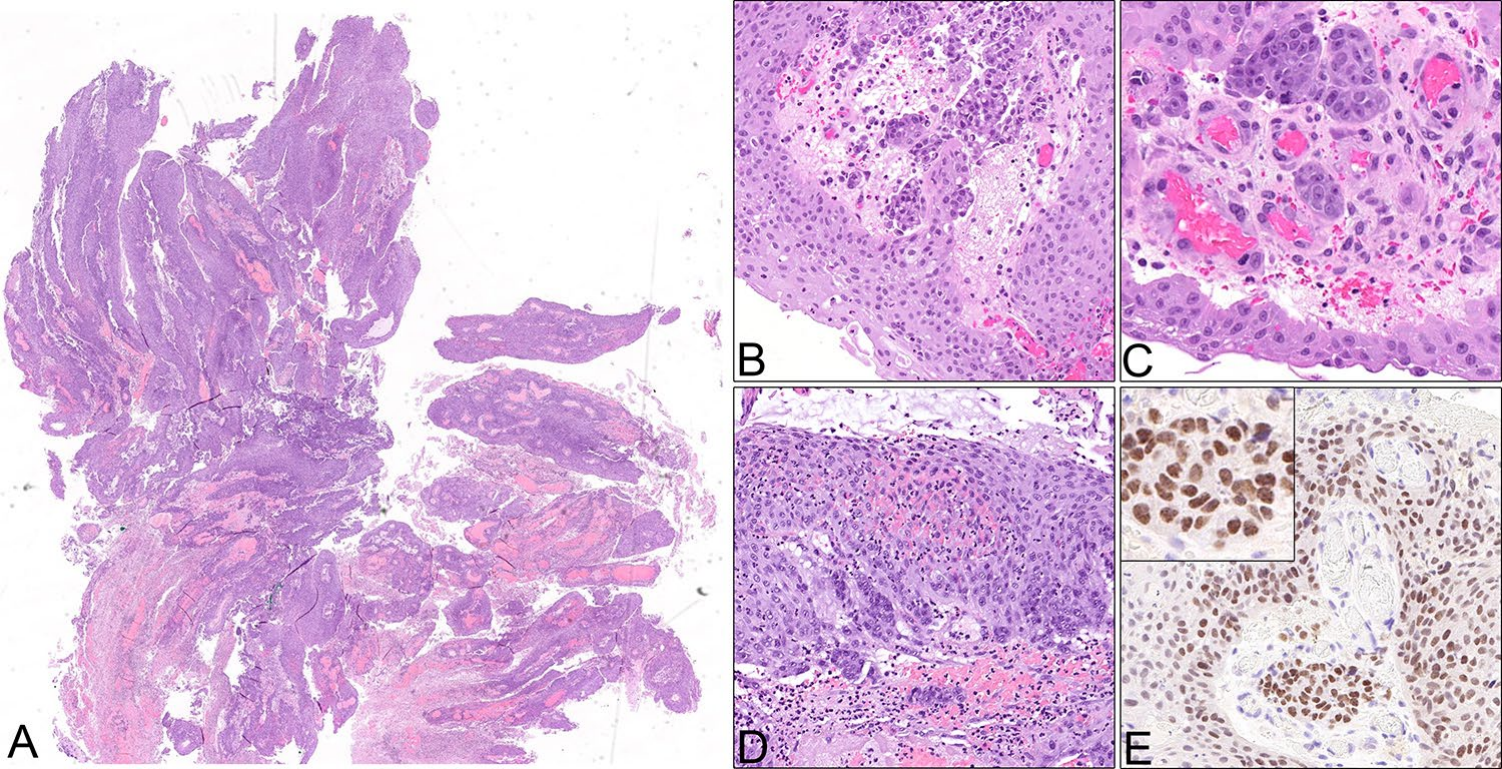
**A**–**D** Histopathology of the tumor: **A** Microscopically, the tumor shows papillomatous-exophytic well-differentiated squamous differentiation (10x), **B**–**C** infiltration of basaloid-appearing, less differentiated cells into the adjacent stroma (20x and 40x), **D** accompanied by prominent granulocytic infiltration (20x). **E** Immunohistochemistry for NUT (C52B1, Rabbit mAb, Cell Signaling Technology) (40x, magnified inset)

The initial biopsies revealed an exophytic-papillomatous (Fig. 3A), partly inverted tumor with squamous differentiation without unequivocal evidence of invasion. Based on the clinical context of a prior tooth extraction with persistent oro-antral fistula, differential diagnostic considerations encompassed prominent reactive inflammatory changes as well as an exophytic-papillomatous, well-differentiated carcinoma. Mucocytes were not detected in the Alcian-Blue-PAS-stain. Therefore, despite abundantly admixed granulocytes, a sinonasal papilloma (which could have aided the diagnosis as a possible precursor lesion) could not be confirmed. In light of the relatively mature squamous differentiation, minimal cytologic atypia and prominent inflammation, a clear diagnosis was hampered. In the second biopsy, small, discohesive collections of epithelial cells infiltrating the stroma with focal transformation into larger, basaloid aggregates without clear demarcation by a basement membrane, militated against the diagnosis of a reactive process (Fig. 3B–D). Additionally, the time course and clinico-radiological features favored a malignant process. The interpretation as a reactive squamous epithelial proliferation was revised with the descriptive diagnosis of an exophytic-papillomatous and partly endophytic growing carcinoma. In the ensuing external pathologic consultation, a diagnosis of a non-keratinizing squamous cell carcinoma (NKSCC) was rendered, assuming that the lesion originated from the sinonasal tract rather than the mucosa of the oral cavity, based on the latest WHO classification of Head & Neck Tumours 5th edition (beta version) [2]. HPV DNA testing as well as EBV-RNA in situ hybridization and p16 immunohistochemistry were negative. In order to address the differential diagnosis of a *DEK::AFF2* fusion-associated carcinoma, molecular profiling was performed using the FoundationOne^®^ Heme test. *DEK::AFF2* fusion-associated carcinomas have been described recently as an emerging entity in the sinonasal tract, with the majority showing a strikingly bland histologic appearance and overlap with so-called low-grade papillary Schneiderian carcinomas [3, 4]. Importantly, the detection of a *DEK::AFF2* gene fusion would allow for more accurate classification and prognostic assessment. Surprisingly, no *DEK::AFF2*, but a *NUT::NSD3* gene fusion was detected, leading to the diagnosis of a NUT carcinoma. A subsequently performed NUT immunohistochemistry (Fig. 3E) showed a matching “speckled type” positivity in the majority of the carcinoma cells (almost 100% in both, basaloid and more differentiated components), corroborating the diagnosis and visualizing the fusion product. In concordance with the morphology lacking mucocytes, no *EGFR* mutation was detected, which are very common in inverted sinonasal papilloma and their carcinoma ex papilloma [5]. The macroscopy of the following left-sided hemimaxillectomy showed the main tumor originating in the maxillary sinus and breaking through the bone into the oral cavity. Together with the neck dissection specimen level I-III the final pathologic tumor staging (according to carcinomas of the nasal cavity and paranasal sinuses) was pT2 pN0 (0/57) L0 V0 Pn1, high-grade, R0 (UICC/TNM 8^th^ edition, 2017). Extensive perineural spread was noted.

### Clinical Follow-up

Adjuvant local radiotherapy was recommended at our multidisciplinary tumorboard. One year after diagnosis and six months after completion of treatment (at the time of the case report submission), the patient showed no evidence of disease, neither clinically nor on PET/CT.

## Discussion

The histological and clinical features of the current case represent a highly unusual constellation. Typical NUT carcinoma is characterized by a more undifferentiated monomorphic morphology with small squamous islets and abrupt keratinization. These features were not present in our case. Nevertheless, the molecular profile, the *NUT::NSD3* gene fusion, has been recurrently described in NUT carcinomas and confirms the diagnosis, especially in association with a squamous phenotype. Accordingly, this case can be regarded as part of the spectrum of NUT carcinomas and emphasizes the importance of considering this differential diagnosis in mature and well-differentiated squamous cell carcinoma. Such atypical features as well as the lack of awareness of this entity suggest an under-diagnosis and -reporting of NUT carcinomas [6]. The partly prominent squamous epithelial differentiation and the growth pattern are highly unusual and to the best of our knowledge have not been described in NUT carcinomas. In this regard, NUT carcinomas are characterized by translocation-associated fusion oncoproteins that interfere with cell differentiation and cell growth. The majority of NUT-fusions involves BRD4 (bromodomain containing protein 4), leading to an epigenetically induced block of cell differentiation and promotion of cellular growth. *NSD3* encodes a histone lysine methyltransferase that binds the extraterminal domain of BRD. In cases harboring the *NUT::NSD3* fusion, this alteration probably leads to similar functional oncogenic consequences. However, as presented in this case, the level of interference with cell differentiation might be different in *NUT::NSD3* fusion than in *NUT::BRD4* fusion [7]. This could explain why *NUT::NSD3* fusion positive carcinomas outside the thorax appear to have a significantly better prognosis than their *NUT::BRD4* positive counterparts [8]. An additional diagnostic challenge are the reactive, inflammatory squamous epithelial changes, which can be prominent after an intervention such as a tooth extraction. The relatively young patient age and the unusual morphology led to the consideration of an HPV-associated carcinoma, which could not be substantiated, as immunohistochemistry for p16 and molecular analysis for HPV DNA were negative. A carcinoma with *DEK::AFF2* gene fusion was considered as the primary differential diagnosis on morphologic grounds. These carcinomas have recently been described and exhibit similar morphologic features to the current case [3, 4]. Importantly, this case presented significant morphological overlap with other head and neck carcinomas. The *NUT::NSD3* gene fusion has recently been described in a subset of thyroid carcinomas without classical features, so that there is a rationale for NUT immunohistochemistry and/or molecular testing in unusual cases. In particular, there is increasing evidence that NUT gene fusions can occur in tumors with different underlying cell types (other than squamoid-like cells), such as thyroid follicle cells. Additional data are needed for accurate classification of these increasingly detected neoplasms [9, 10]. The concept of tumoral-mucosal fusion as a potential pitfall of processes underlying the surface mucosa is recognized in minor salivary gland neoplasia [11]. However, the observation that the majority of bland squamous cells in the mucosa were NUT IHC positive in our case, suggests that maturation may be involved. This case further demonstrates that highly sensitive and specific NUT immunohistochemistry is useful in identifying cases with unusual morphology, thus enabling accurate classification.

Future studies on larger numbers of cases are needed for comparing the biological behavior and other features of “differentiated NUT carcinoma” with the classical type.

## Data Availability

Not Applicable.
